# First Case Report of Fulminant Hepatitis After Laparoscopic Sleeve Gastrectomy Associated with Concomitant Maximal Therapeutic Dose of Acetaminophen Use, Protein Calorie Malnutrition, and Vitamins A and D, Selenium, and Glutathione Deficiencies

**DOI:** 10.1007/s11695-020-04999-y

**Published:** 2020-10-22

**Authors:** Alyaa Abusabeib, Walid El Ansari, Jassim Alobaidan, Wahiba Elhag

**Affiliations:** 1 Department of Bariatric Surgery/Bariatric Medicine, Hamad General Hospital, 3050 Doha, Qatar; 2 Department of Surgery, Hamad General Hospital, 3050 Doha, Qatar; 3 College of Medicine, Qatar University, Doha, Qatar; 4 Schools of Health and Education, University of Skovde, Skövde, Sweden

**Keywords:** Liver failure, Fulminant hepatitis, Paracetamol toxicity, Malnutrition, Sleeve gastrectomy, Vitamin A deficiency, Selenium deficiency, Glutathione deficiency

## Abstract

Nonalcoholic fatty liver disease (NAFLD) is increasingly being linked to obesity. Although laparoscopic sleeve gastrectomy (LSG) is effective for weight loss that can ultimately resolve NAFLD, an initial transient deterioration of liver functions could be observed during the first few months post-operatively, after which a subsequent improvement of the liver functions might occur. Rapid weight loss, nutritional deficiencies, and protein malnutrition can all contribute to hepatic dysfunction and can affect the metabolism of medications such as acetaminophen leading to more insult to a compromised liver. We report acute liver failure after LSG associated with protein calorie malnutrition, multiple nutritional deficiencies in addition to concomitant use of therapeutic doses of acetaminophen. Treatment with N-acetylcysteine, and replacement of deficient multivitamins and trace elements resulted in significant improvement in liver functions.

## Background

Nonalcoholic fatty liver disease (NAFLD) is due to an increased deposition of triglycerides into the hepatocytes to around 5% of liver weight [1]. NAFLD ranges from simple steatosis (relatively benign) to severe nonalcoholic steatohepatitis (NASH, can lead to cirrhosis and hepatocellular carcinoma) [1]. NAFLD is increasingly observed as a complication of obesity and also as part of the metabolic syndrome.

Laparoscopic sleeve gastrectomy (LSG) is common and effective for weight loss, acting through restrictive and hormonal mechanisms. Although such weight loss can ultimately slow/stop the progression of or resolve the NAFLD [2], however, various extents of hepatic dysfunction could be encountered post bariatric surgery (BS) due to a range of factors.

For instance, after BS, an initial transient deterioration of liver functions to the extent of possible liver failure is observed during the first few months especially after Roux-en-Y gastric bypass, after which a subsequent improvement of the liver functions might occur [3]. Such deterioration could be multifactorial: rapid weight loss leading to enhanced lipolysis and increased release of endogenous free fatty acids from adipose deposits which may in turn increase the risk of liver fibrosis [2]; changes in gut microbiota may contribute to hepatic dysfunction; protein calorie malnutrition/starvation leads to autophagy resulting in liver cell necrosis; and dehydration results in poor blood supply to the liver [4, 5]. In addition, as part of the nutritional deficiencies encountered after BS, low vitamin D levels can be associated with severe histologic changes in NAFLD; low vitamin A was linked to progression of NAFLD; and selenium deficiency is associated with decreased protection against oxidative stress [6–9]. Amidst such collective insults, common medications could prove an additional burden on an already compromised liver [10].

We report a fulminant hepatic failure 2 months after LSG accompanying protein calorie malnutrition and vitamins A, D and selenium deficiencies, associated with concomitant maximal therapeutic dose of acetaminophen use and possible glutathione deficiency.

## Case Report

A 26-year-old Qatari female presented to the emergency room at our institution (Hamad Medical Corporation, largest tertiary care center in Qatar) on 1 Nov. 2019, complaining of a 3-week nausea, repeated vomiting and severe upper abdominal pain radiating to the back, with no aggravating factors and minimally relieved by paracetamol. She had normal bowel habits but decreased frequency and amount of urine. The patient reported fatigue and bilateral numbness episodes of the fingertips that resolved spontaneously, but no fever, skin lesions, or skin color change. She had no other sensory complaints weakness, dizziness, or visual complaints. Past history was remarkable for obesity class 3 (BMI 40 kg/m^2^) and benign intracranial hypertension controlled with acetazolamide. Two months earlier, weighing 95 kg (BMI 40 kg/m^2^), she underwent LSG that reduced her weight to 79 kg, and was off acetazolamide. Post-LSG, she tolerated pureed but not soft diet because of nausea. She denied blood transfusions, recent travel, smoking or alcohol consumption, contact with sick persons, but reported nonadherence to the prescribed multivitamins and high protein supplements.

**Fig. 1:**
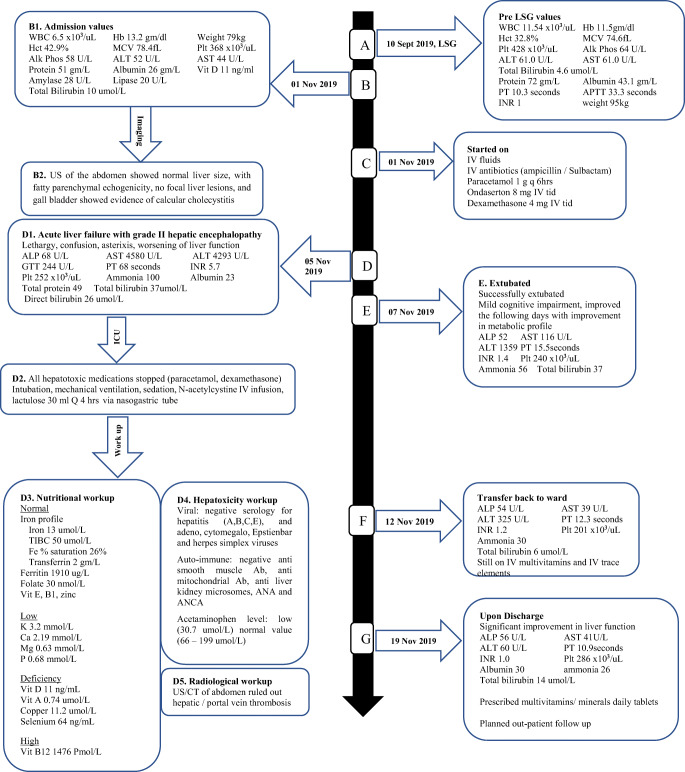
Timeline and sequence of events. *LSG* Laparoscopic sleeve gastrectomy, *US* ultrasound. Reference values: *WBC* white cell count (4–10 × 10^3^/uL), *Hct* hematocrit (36–46%), *MCV* Mean corpuscular volume (83–101 fL), *Hb* hemoglobin (12–15 g/dl), *Plt* platelet (150- 400 × 10^3^/uL), *Alk Phos* alkaline phosphatase (35–104 U/L), *ALT* alanine aminotransferase (0–33 U/L), *AST* aspartate aminotransferase (0–32 U/L), total bilirubin (0-21umol/L), total protein (66-87 g/L), albumin (35–52 g/L), PT (9.7–11.8 s), APTT (24.6–31.2 s), INR 1, amylase (13–60 U/L), lipase (13–53 U/L), ammonia (11–51 umol/L), folate (10.4–42.4 nmol/L), iron profile: iron (6–35 umol/L), *TIBC* total iron binding capacity (45–80 umol/L), Fe % saturation (15–45%), transferrin (2–3.6 g/L), ferritin (12–114 μg/L), vitamin A (1.05–2.09 umol/L), vitamin B12 (133–675 pmol/L), zinc (10.1–16.8 umol/L ), selenium (70–150 ng/ml), vitamin D (35–88 ng/mL), copper (11.8–22.8 umol/L), *K* potassium (3.5–5.1 mmol/L), *Ca* calcium (2.2–2.5 mmol/L), *Mg* magnesium (0.66–1.07 mmol/L), *P* phosphorus (0.81–1.45 mmol/L), *ANA* antinuclear antibody, *ANCA* antineutrophil cytoplasmic antibodies

Upon examination, she was vitally stable, oriented, with clear chest and normal cardiovascular and central nervous system, normal bowel sounds, but right upper abdominal quadrant tenderness. Liver enzymes were mildly deranged (Fig. 1 B1), US of the liver showed fatty parenchymal echogenicity and calcular cholecystitis (Fig. 1 B2).

The acute surgery team admitted her and commenced treatment (Fig.1C). Four days later, with more abdominal pain, unimproved nausea and vomiting, and acute liver failure (ALF) with grade II hepatic encephalopathy (Fig.1 D1), she was transferred to intensive care unit (Fig.1 D2), where nutritional, hepatoxic, viral serologies, auto-immune profiles work ups were undertaken, as well as CT and US of the abdomen (Fig. 1 D3, D4, and D5). The gastroenterology team considered liver transplant; however, her liver function gradually improved over the following 2 days, and she was extubated (Fig. 1E).

She was transferred to the ward on 12 November (Fig.1F), followed up by a multidisciplinary team, was gradually tolerating soft mechanical diet, remained asymptomatic and showed significant improvement in liver function. She was discharged on 19 November 2019 (Fig. 1G).

## Discussion

We report a patient with history of mild derangement in liver function on the day prior to her LSG. Two months post-LSG, she presented at our hospital (index admission) and was diagnosed as calculus cholecystitis. At this stage, she had mildly deranged liver and was admitted by the general surgery team and started on treatment. Our first encounter with her was on day 5 of this index admission, where she was drowsy with lethargy, moderate confusion, asterixis, and severe transaminitis. Hence, we diagnosed acute liver failure.

**Fig. 2:**
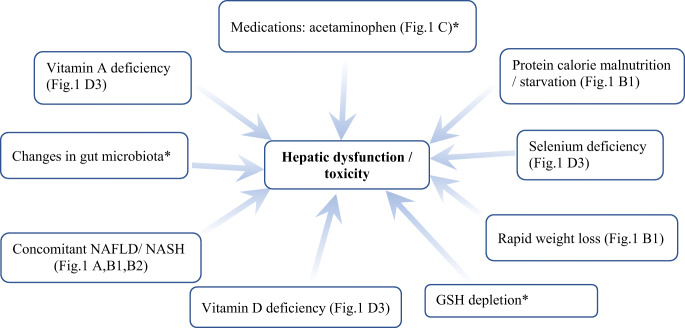
Multiple concomitant risk factors for liver toxicity after bariatric surgery. Capital letters within brackets in the boxes refer to the evidence available to bariatric team for suspicion of the given cause (from Fig. 1) to the liver toxicity encountered in our patient. *GSH* glutathione, *NAFLD* nonalcoholic liver disease, *NASH* nonalcoholic steatohepatitis. * indicates speculated factors, with no evidence available to the bariatric team for its direct effects on hepatic dysfunction/toxicity in the current patient

Liver failure post bariatric surgery has been described after some of the “older” procedures, e.g., jejunoileal bypass and biliopancreatic diversion, but is rare in modern BS, e.g., LSG [11]. Nevertheless, ALF is encountered post BS due to a range of factors [5], as depicted in Fig. 2 for our patient.

As a procedure, LSG seems not directly implicated in ALF. On the contrary, BS generally has positive impacts on liver enzymes and histology. Particularly, LSG could be beneficial in decreasing the systemic oxidative stress observed with obesity, with positive prognosis for NAFLD/NASH patients [12, 13]. A meta-analysis (15 studies, 766 liver biopsies) observed significant improvements in the NAFLD components, namely, liver steatosis, steatohepatitis, and fibrosis in 91.6%, 81.3%, and 65.5% of patients, respectively, and complete resolution among 69.5% of patients for nonalcoholic steatohepatitis after BS [14].

As for rapid weight loss after LSG, within the previous 7 weeks pre-admission, our patient lost 16 kg (Fig. 1. B1), amounting to 2.2 kg per week. Rapid weight loss (> 1.6 kg per week) may increase visceral free fatty acids and proinflammatory cytokines, which increase the risk of liver fibrosis [2]. Likewise, rapid weight loss may also precipitate mild lobular hepatitis [15]. In addition, the associated protein malnutrition and resultant rapid mobilization of intra−/extrahepatic fat stores during weight loss could aggravate preexistent liver steatosis in these patients [15]. Collectively, such mechanisms can lead to ALF as observed in the current case.

Related to the protein malnutrition and rapid weight loss is the alteration of micronutrient absorption after BS. Our patient had vitamins A, D and selenium deficiencies (Fig.1 D3). In terms of vitamin A, declining circulating and hepatic retinol levels are associated with progression of NAFLD to NASH, cirrhosis, and cancer [8]. Likewise, low vitamin D was associated with more severe histologic changes in NAFLD [6]. High selenium levels were associated with increased prevalence of NAFLD [16]; and conversely, selenium deficiency was associated with increased liver damage in experimental NAFLD models, where zinc and selenium co-supplementation improved the serum biochemical parameters such as liver enzymes and lipid profile with reductions in fat granule accumulation in the liver and liver size [9]. Such conjoint micronutrient deficiencies could have contributed to the patient’s acute liver failure.

As for exogenous insults, some medications commonly prescribed to bariatric patients can contribute to liver deterioration. Following most BS types, patients avoid nonsteroidal anti-inflammatory drugs given the increased risk of such drugs for gastrointestinal ulceration. Thus, acetaminophen is among the remaining non-narcotic analgesics suitable for such patients [7]. However, baseline nutritional status might predispose to more severe acetaminophen liver injury [10]. Among patients with acetaminophen-associated ALF, those with prior BS had higher INR, lower serum albumin, and a trend toward a higher coma grade [7]. In agreement, our patient was on maximal therapeutic dose of acetaminophen and had high INR (6), low albumin (27 g/L), and moderate cognitive impairment. Her blood acetaminophen level was low (Fig.1 D4); hence, therapeutic doses could prove toxic to a liver already compromised by a range of factors as outlined above [12].

As regards to GSH, it plays a key role in the protection of the liver by detoxification of both endogenous and exogenous toxic metabolites [17]. With acetaminophen, hepatotoxicity is not caused by the drug itself, but by its reactive intermediate N-acetyl-p-benzoquinone imine (NAPQI) [18]. It is unclear whether post-BS patients have lower intrahepatic GSH stores, which limits NAPQI detoxification, causing liver injury, as seen in patients with malnutrition or chronic alcohol abuse [19]. N-acetylcysteine (NAC) replenishes intracellular GSH levels [20], thus enhancing the liver to remove toxic metabolites. There are no facilities to measure GSH at our institution, and we cannot confirm GSH deficiency in our patient; however, administration of NAC resulted in positive response with significant liver function improvements within a few days.

In terms of preexisting health issues, we encountered a hospitalized, rather sick patient. Hence, we did not undertake invasive liver biopsy and cannot confirm whether she had NAFLD at this stage. Nevertheless, we are also unable to confirm that she did not have preexisting NAFLD for three reasons: her hospital records showed mild derangement of liver function on the day prior to her LSG; NAFLD can still be observed despite normal serum liver enzyme levels [21]; and index admission US abdomen showed normal liver size but fatty parenchymal echogenicity.

## Conclusion

Despite the many positive outcomes of LSG, hepatic dysfunction, fulminant hepatitis, and liver failure can sometimes be observed due to complex interlacing factors. Rapid weight loss, protein malnutrition, macro-/micronutritional deficiencies, and the generated oxidative stress could collectively contribute to hepatic dysfunction and negatively affect the metabolism of common medications such as therapeutic acetaminophen doses leading to additional insult to a liver already compromised by NAFLD/NASH as outlined. Close monitoring and multidisciplinary follow-up of patients after bariatric surgery are recommended for prevention, early detection, and management of such conditions, along with the cautious use of acetaminophen in vulnerable patients.
